# Early-Stage Ice Detection Utilizing High-Order Ultrasonic Guided Waves

**DOI:** 10.3390/s24092850

**Published:** 2024-04-29

**Authors:** Regina Rekuvienė, Vykintas Samaitis, Audrius Jankauskas, Abdolali K. Sadaghiani, Shaghayegh Saeidiharzand, Ali Koşar

**Affiliations:** 1 Prof. K. Barsauskas Ultrasound Research Institute, Kaunas University of Technology, K. Barsausko St. 59, LT-5142 Kaunas, Lithuania; 2 Sabanci University Nanotechnology and Application Centre (SUNUM), Sabanci University, Istanbul 34956, Turkey; 3 Faculty of Engineering and Natural Sciences (FENS), Sabanci University, Istanbul 34956, Turkey; 4 Center of Excellence for Functional Surfaces and Interfaces for Nano-Diagnostics (EFSUN), Sabanci University, Istanbul 34956, Turkey

**Keywords:** ultrasonic guided waves, high-order modes, antifreeze coatings, ice binding proteins

## Abstract

Ice detection poses significant challenges in sectors such as renewable energy and aviation due to its adverse effects on aircraft performance and wind energy production. Ice buildup alters the surface characteristics of aircraft wings or wind turbine blades, inducing airflow separation and diminishing the aerodynamic properties of these structures. While various approaches have been proposed to address icing effects, including chemical solutions, pneumatic systems, and heating systems, these solutions are often costly and limited in scope. To enhance the cost-effectiveness of ice protection systems, reliable information about current icing conditions, particularly in the early stages, is crucial. Ultrasonic guided waves offer a promising solution for ice detection, enabling integration into critical structures and providing coverage over larger areas. However, existing techniques primarily focus on detecting thick ice layers, leaving a gap in early-stage detection. This paper proposes an approach based on high-order symmetric modes to detect thin ice formation with thicknesses up to a few hundred microns. The method involves measuring the group velocity of the S_1_ mode at different temperatures and correlating velocity changes with ice layer formation. Experimental verification of the proposed approach was conducted using a novel group velocity dispersion curve reconstruction method, allowing for the tracking of propagating modes in the structure. Copper samples without and with special superhydrophobic multiscale coatings designed to prevent ice formation were employed for the experiments. The results demonstrated successful detection of ice formation and enabled differentiation between the coated and uncoated cases. Therefore, the proposed approach can be effectively used for early-stage monitoring of ice growth and evaluating the performance of anti-icing coatings, offering promising advancements in ice detection and prevention for critical applications.

## 1. Introduction

The detection of ice poses a long-standing challenge, especially in the renewable energy and aviation sectors, where it can significantly affect aircraft performance during flight and lead to reduced wind energy production. Numerous ice-related incidents involving aircraft have been reported, underscoring the critical importance of understanding the dynamic effects of ice on aircraft performance [1]. Notable recent incidents include West Wind Aviation Flight 282 in Canada in 2017 and the Bek Air Flight 2100 crash in Kazakhstan in 2019, both of which resulted in a significant loss of altitude shortly after take-off due to ice contamination, leading to 15 fatalities [2,3]. Ice buildup on aircraft airframes or wind turbine blades alters their surface roughness, creates ice protuberances, and induces local airflow separation [4,5]. Consequently, aircraft experience a decrease in the maximum lifting coefficient and the slope of the lifting curve, along with an increase in drag and the critical stall speed [6]. Wing icing diminishes the stall angle of attack, while tail-plane icing can lead to tail-plane stalling and an increase in stick force, complicating aircraft control. Additionally, asymmetric ice formation can introduce additional rolling moments, limiting lateral control [7]. For wind turbine blades, icing can lead to a reduction in power production by up to 50% of the annual output [8]. In addition, it can increase wear on turbine components due to changes in aerodynamic properties, resulting in a shorter lifespan of the structure and higher maintenance costs [5].

Ice formation can arise from various sources, including supercooled water droplets, pre-takeoff contamination, and encounters with high-concentration ice crystals during flight [6]. To address the effects of icing, multiple approaches have been proposed. These include chemical solutions that lower the freezing point, pneumatic systems that inflate boots to break the ice layers, and heating systems that warm the lifting surfaces of an aircraft [9,10,11,12]. However, these solutions are considered costly, leading to increased fuel consumption while protection is limited to certain parts of the aircraft [13]. To optimize the cost-effectiveness of ice protection systems, it is essential to have reliable information about current icing conditions that can trigger their activation.

Icing detection systems can be based on computational fluid dynamics, which predict the shape and severity of the ice under various flight conditions. Alternatively, they can rely on direct measurements of ice accretion on the body or parts of the wind turbine blades [7,9]. Among the direct ice accretion detection techniques, impedance and capacitance, vibration, fiber optic, and ultrasonic sensors are most widely adopted. For example, Schlegl et al. [14] used impedance sensors for ice detection based on differences in electrical properties between air, water, and ice. Zheng et al. [15] demonstrated that capacitance measurement can well reflect ice growth, while impedance spectra can differentiate between ice types. Mader et al. [16] used piezoceramic sensors and measured impedance and phase to estimate changes in natural resonant frequencies and their relation to icing. Zou et al. [17] used optical fiber sensors with oblique end-faces to detect ice based on optical intensity, while Ikiades et al. [18] demonstrated a real-time approach to detecting ice by measuring the optical intensity of backscattered and reflected light. Liu et al. [19] measured the frequency-dependent attenuation of ultrasound to characterize ice types. Similarly, Fuleki [20] measured the reflection amplitude of ultrasonic waves to determine whether the surface is surrounded by air, ice, or water. Finally, Sohail et al. [21] employed acoustic emission to detect the freezing of water sprayed on supercooled substrates.

Although most of the techniques proved to be effective, they are mostly localized and cannot cover large areas of an aircraft. The use of ultrasonic guided waves offered notable advancements over the other existing techniques, allowing for the integration of sensors to the structure of the aircraft without compromising the aerodynamics and allowing for the detection of icing up to a few meters away from the single sensor position. Rose et al. [22] analyzed guided wave interactions with icing on an aluminum sheet using shear horizontal waves and demonstrated the monitoring of icing formation. The authors demonstrated changes in the time-of-flight, cut-off frequency, and magnitude of the shear horizontal mode in case of rime, glaze, and mixed ice at different ice thicknesses [22]. Mendig [23] monitored ice accretion on the NACA 0012 profile using fundamental symmetric modes. The authors found a decrease in the group velocity of the S0 mode with an increasing thickness of the ice layer. Moll et al. [24] analyzed the formation of ice on a glass fiber plate submerged in water. The authors demonstrated that at low frequencies, ice induces significant amplitude changes in the fundamental asymmetric mode. Similar effects were observed for the symmetric mode at higher frequencies. In another study, Memmolo et al. [25] analyzed sprayed icing on a glass fiber panel and demonstrated the signal energy, phase velocity, and phase shift due to the length and thickness.

Recent advances in guided wave icing detection have demonstrated that various parameters such as signal energy, velocity, cutoff frequency, or phase can serve as indicators to detect icing and monitor its thickness. However, most of the studies presented in the literature focus on relatively thick ice layers, typically ranging from 2 to 15 mm in thickness. It is expected that such thick ice layers, especially those formed layer by layer with water spraying, may exhibit more pronounced changes in guided wave propagation. Furthermore, while changes in magnitude due to icing are evident, such measurements are sensitive to various environmental factors, such as structural vibrations during operation. Inspired by recent findings indicating that high-frequency guided waves can be utilized for early-stage ice detection with thicknesses below 0.5 mm [5,26], this paper proposes an approach based on high-order symmetric modes to detect thin ice formation with thicknesses of up to a few hundred microns. To achieve this objective, we employed a group velocity measurement in the high-order symmetrical mode and proposed a frequency-dependent group velocity reconstruction technique based on bandpass filtering of acquired signals propagated on samples with or without ice. For the implementation of thin ice layers, the natural humidity in the air was utilized to form the ice layers on the samples. The technique was experimentally tested on 1.5 mm thick copper samples with different surface conditions. One sample served as a reference and had an original uncoated surface, while the other possessed a superhydrophobic multiscale coating (SHMC) that decreases ice adhesion strength and prevents ice formation [27,28]. Experimental investigations were conducted on both samples at surface temperatures ranging between +25 °C and −15 °C. The results demonstrated the successful detection of ice formation and illustrated the potential for monitoring the growth of the ice layer.

## 2. Object under Inspection and Selection of Wave Mode

**Figure 1:**
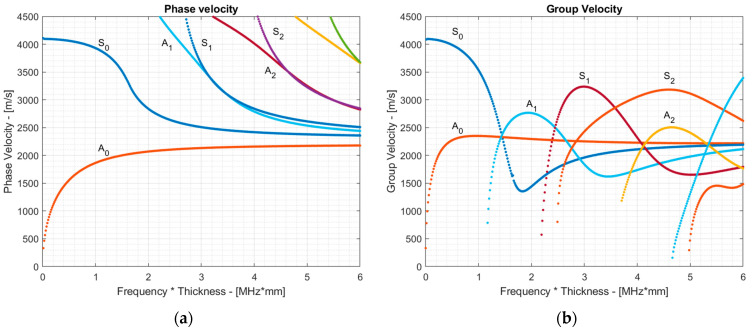
Dispersion curves in a 1.5 mm copper plate: (**a**) phase velocity; (**b**) group velocity.

In this study, we explored icing on 1.5 mm thick copper plates with dimensions of 45 mm × 45 mm. The following material properties are used to describe the material: Youngs modulus *E* = 130 GPa, Poisson ratio *ν* = 0.34, and density *ρ* = 8960 kg/m^3^. To explore waves propagating in the structure, the dispersion curves of guided waves were calculated using the semi-analytical finite element technique (SAFE) in the frequency range up to 6 MHz·mm. The resulting estimated phase and group velocity dispersion curves are presented in Figure 1. The results presented in the figure below indicate that a fundamental mode zone existed up to 1.2 MHz·mm, and the higher-order zone began above this point.

**Figure 2:**
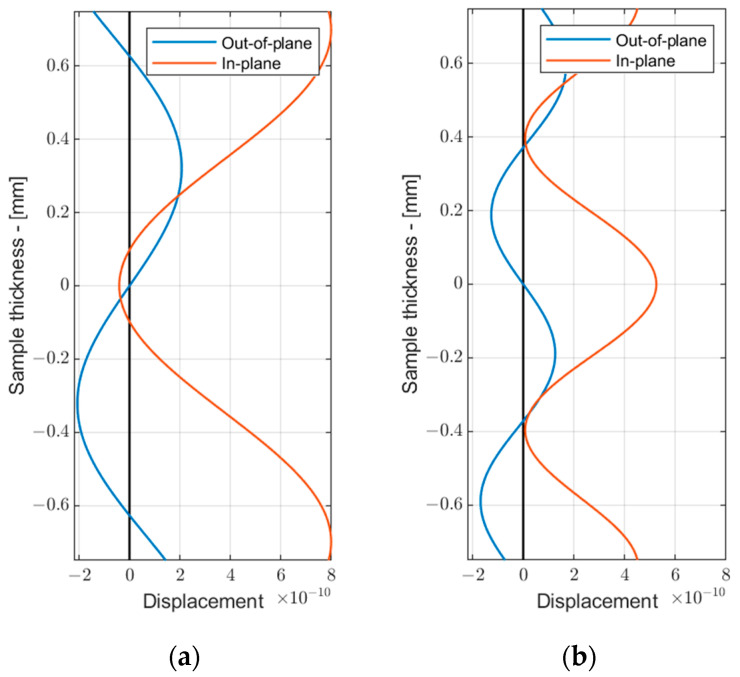
The in-plane and out-of-plane displacements for S_1_ mode at 3 MHz·mm (**a**) and S_2_ mode at 5.1 MHz·mm (**b**).

To select the mode suitable for inspection, the group velocities of the modes and their through-thickness displacements must be considered. The results presented in Figure 1b suggested that the S_0_, A_1_, S_1_, and S_2_ modes were potential candidates for inspection, as each of them possessed the highest group velocity at a specific frequency, allowing them to arrive first to the receiver and thus making the signal analysis easier. In this paper, our focus was on high-order modes that could potentially offer better sensitivity for early-stage ice detection, as presented by other authors [22]. This suggestion is confirmed in the following Section 3, which demonstrates the plots of group velocity change for both the S_0_ and S_1_ modes over the ice thickness, confirming the enhanced resolution of the high-order modes compared to the fundamental ones. Additionally, due to access constraints to the surface of the coated sample, we mainly considered symmetrical modes that could be excited by mounting the probe to the edge of the sample. Therefore, the S_0_ and A_1_ modes were not further considered. The choice between the high-order modes such as S_1_ and S_2_ was made based on the through-thickness displacements for these modes, as depicted in Figure 2. The displacement profiles for these modes were plotted at frequencies corresponding to the maximum group velocity value—at 3 MHz·mm for the S_1_ mode and 5.1 MHz·mm for the S_2_ mode. To ensure the comparability of displacement magnitudes between different modes, the total energy of both the S_1_ and S_2_ modes was kept constant throughout the calculations.

As seen from the results in Figure 2, the S_1_ mode exhibited slightly larger out-of-plane displacements, suggesting that it may be more sensitive to ice loading. Additionally, it demonstrated high group velocity dispersion, indicating that even small changes in the overall thickness due to ice accretion will result in notable group velocity changes. Based on these findings, the S1 mode was selected for further analysis. The selection of the inspection frequency for the S_1_ mode was influenced by its group velocity dispersion. While one might suggest that exciting the S_1_ mode at the zero-group velocity point at 2 MHz·mm would enhance the sensitivity to ice loading, co-existing modes at this inspection frequency may overlap with the S_1_ mode, leading to complicated signal analysis. Therefore, in this study, we opted for a 3 MHz·mm inspection frequency, sacrificing resolution to simplify the signal analysis.

## 3. Guided Wave Propagation on Structures with Ice

**Figure 3:**
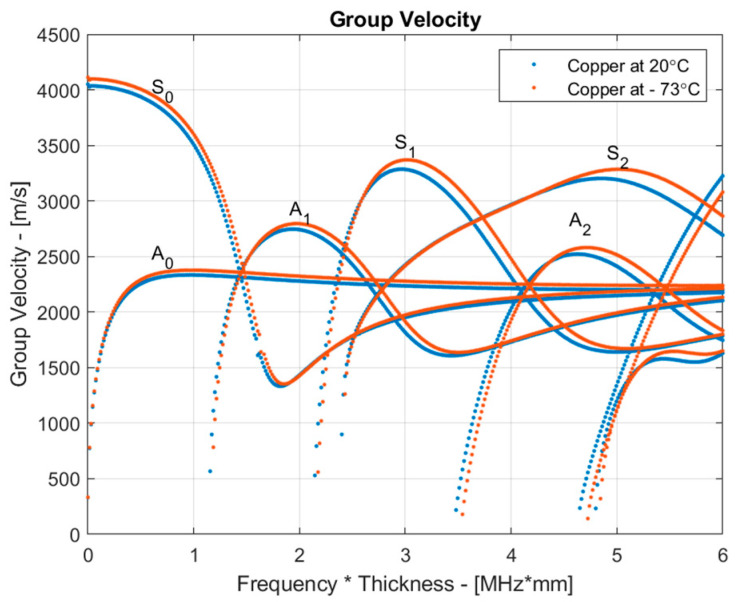
Group velocity dispersion curves in a 1.5 mm copper plate at 20 °C and −73 °C.

It is well known that the group velocity of guided waves is temperature-dependent and increases with decreasing temperature [29]. To illustrate this phenomenon, dispersion curves for group velocity were calculated for a 1.5 mm copper plate at room temperature (20 °C) and at −73 °C. For this analysis, we accounted for temperature-induced changes in Young’s modulus and Poisson’s ratio while considering density changes as negligible within this temperature range. The following material properties of copper were utilized [30]: *E* = 128.1 GPa, *ν* = 0.35 at 20 °C and *E* = 132.56 GPa, *ν* = 0.346 at −73 °C. The dispersion curves were computed using the semi-analytical finite-element (SAFE) method and are presented in Figure 3. The results depicted in Figure 3 confirm that with decreasing temperature, the velocity of the S1 mode increased. It is important to note that these estimations only considered temperature effects and did not account for the formation of the ice layer.

**Figure 4:**
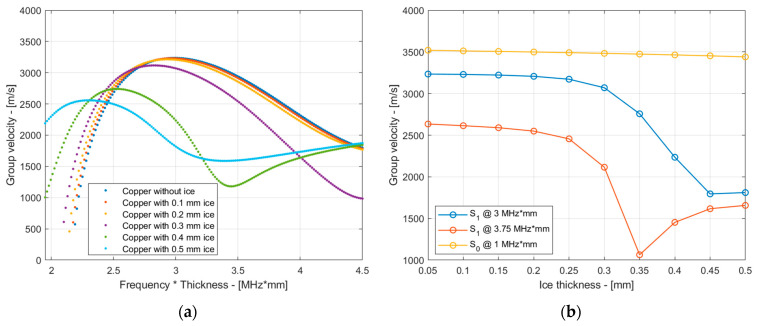
Comparison of the group velocity dispersion curve for the S_1_ mode at varying thicknesses of ice up to 0.5 mm (**a**) and the variation in the group velocity of the S_1_ mode at 3 MHz·mm and 3.75 MHz·mm and S_0_ mode at 1 MHz·mm as a function of ice thickness (**b**).

In the presence of an ice layer, the structure becomes two-layered, resulting in an increase in the total thickness of the material. A symmetry of about half the thickness of the structure is no longer maintained, and the modes cannot be referred to as symmetric and asymmetric. However, for consistency, we will continue to refer to the modes by their initial names hereafter. Figure 4a depicts a comparison of the group velocity dispersion curve of the S_1_ mode with varying thicknesses of ice up to 0.5 mm. Similarly, Figure 4b shows the group velocity of the S_1_ mode at 3 MHz·mm and 3.75 MHz·mm as a function of ice thickness. In the latter figure, we additionally included the group velocity variation in the ice thickness for the S_0_ mode at 1 MHz·mm to illustrate the enhanced sensitivity of high-order modes such as S_1_ for early-stage ice detection. For these calculations, we assumed that the properties of copper were the same as those used in the previous section. The properties of ice were assumed to be as follows: *E* = 9.3 GPa, *ν* = 0.325, and *ρ* = 900 kg/m^3^. These properties correspond to glaze/mixed ice [22,31].

From the results presented in Figure 4, it follows that the group velocity of the S_1_ mode decreased by around 15 m/s per 0.1 mm of ice accretion at 3 MHz·mm and 50 m/s per 0.1 mm of ice at 3.75 MHz·mm for ice thicknesses up to 0.2 mm. A 1.4 ÷ 7.2 µs delay time can be expected with 0.1 ÷ 0.2 mm ice in the case of a 1 m wave travel distance. As the thickness of the ice increased from 0.3 mm to 0.5 mm, the changes in the group velocity became more pronounced. However, the group velocity response, as seen in Figure 4b, became non-monotonic, and detecting such high-order modes became increasingly challenging as their group velocity approached that of other coexisting modes, potentially leading to overlap in the time domain. It can be observed that as the thickness of the ice increased, the group velocity dispersion curve of the S_1_ mode tended to shift to the left towards lower values of the *f* × *d* product. Consequently, the inspection frequency can be lowered accordingly. For instance, frequencies between 2.25 MHz·mm and 2.5 MHz·mm may be suitable for ice thicknesses of 0.4 mm and 0.5 mm. Meanwhile, employing the detection method utilizing the S_1_ mode at an inspection frequency of 3 MHz·mm is potentially suitable for detecting very thin ice in the early stages, below 0.3 mm.

## 4. Icing Detection Approach

Ice formation can be detected by employing various parameters of guided waves, including signal magnitude, group velocity, attenuation, or cut-off frequency. In this study, we focused primarily on the measurement of the group velocity, which tends to change with the increasing thickness of the ice layer, as demonstrated in the previous section. Additionally, it is expected that changes in the magnitude will be observed, as the ice layer introduces additional scattering of the propagating waves. The measurement approach for the magnitude and group velocity used in this paper can be summarized by the following steps:

1. The signal propagated through the sample, with or without ice, is filtered using a Gaussian bandpass filter with a central frequency equal to the mode of interest. Consequently, the filtered signal, denoted as $u_{f_{D}}\left(t\right)$ is obtained.
2. The magnitude of the filtered signal is estimated according to the following equation:
  (1) $$U_{M}=\underset{t}{max}\left[u_{f_{D}}\left(t\right)\right].$$
3. The time-of-flight $t_{L}$ of the received signal is estimated using the predefined threshold level L according to the following:
  (2) $$t_{L}=\arg\left\{\underset{t}{min}\left[u_{f_{D}}\left(t\right)>L\right]\right\}.$$
  As the S_1_ mode possesses the highest group velocity within the selected frequency range, it is expected to arrive first at the sensor. Therefore, velocity measurement using the threshold level L is assumed to be sufficient for time-of-flight estimation.
4. The group velocity is estimated for the known propagation distance $L_{b}$:
  (3) $$L_{b}/\left(t_{L}-\Delta t_{\mathrm{corr}}\right),$$
  where $\Delta t_{\mathrm{corr}}$ is the time-of-flight correction coefficient estimated during the calibration of the equipment.

Using the abovementioned approach, it is possible to estimate the dependencies of the group velocity and magnitude of the S_1_ mode as a function of temperature and ice formation. When using conventional probes to excite higher-order modes, co-existing modes are inevitable. Therefore, it becomes important to reconstruct the group velocity dispersion curves from the experimental measurements in order to identify all the existing modes and their velocities. In conventional measurements where surface scanning of the sample is available, this can be achieved using 2D FFT, which transforms the time-of-flight vs. distance measurements into the group velocity vs. the frequency domain. Alternative approaches that require fewer measurement points, such as those based on signal filtering and zero-crossing estimation, are also available [32]. In our case, we used sensors located at opposite edges of the samples. To reconstruct the dispersion curves from a single measurement with a fixed propagation distance, we employed an approach based on filtering the signal using different bandpass filters and transforming it into the group velocity domain, as described below:

1. The set of central frequencies for the bandpass filters is defined as $\left[f_{1},f_{2},\ldots ,f_{n}\ldots ,f_{N}\right]$, covering the frequency range of interest.
2. The filter function is selected. In our case, we used a Gaussian filter.
3. The filter function $H_{f_{n}}\left(f\right)$ is generated for the first central frequency *f_n_*, where *n* = 1.
4. The acquired signal $u\left(t\right)$ is filtered using the Fourier transform:
  (4) ufnt=Re⁡FT−1⁡FT⁡ut⋅Hfnf
  where Re denotes the real part, and FT denotes the Fourier transform.

1. The time axis of the filtered signal is transformed into a group velocity $c_{gr}$ axis, considering that the propagation distance $L_{b}$ is known:
  (5) $$u_{f_{n}}\left(t\right)\Rightarrow u_{f_{n}}\left(c_{gr}\right)=u_{f_{n}}\left(L_{b}/t\right).$$
2. The obtained dependence of group velocity on frequency is recorded in the output array for the first central frequency:
  (6) $$B_{f,c_{gr}}\left(f_{n},c_{gr}\right)=u_{f_{n}}\left(c_{gr}\right).$$
3. Steps 3–6 are repeated for other central frequencies defined until the entire frequency range is covered.

As a result, the group velocity as a function of frequency can be obtained similarly to the 2D FFT, albeit using only a single measurement.

## 5. Samples and Anti-Icing Coatings

Coatings on substrate materials can create anti-icing surfaces to prohibit ice accumulation [27]. In this study, we explored two group of samples, coated and uncoated, to monitor changes in acoustic parameters during freezing processes and investigate the differences between the coated and uncoated cases. The coated samples comprised two main layers: a substrate layer and a coating layer. Copper was selected as the substrate layer for several reasons. The primary factors include its compatibility with antifreeze coatings, good thermal conductivity comparable to silver, and well-established acoustic and mechanical properties. These properties enable a more precise estimation of the dispersion characteristics and the simulation of wave propagation regularities. In our experiments, copper plates measuring 4.5 cm × 4.5 cm with thicknesses of 1.5 mm were used. The coating layer was a relatively thin superhydrophobic multiscale coating (SHMC) layer with a thickness ranging from 1 µm to 5 µm. The samples used in this study are summarized in Table 1.

A metal–organic framework (MOF)-based functionalized SHMC coating with an apparent contact angle (CA) value exceeding 171°, a rolling angle of <5°, and a contact angle hysteresis of <3° was immobilized on the oxygen-plasma-pre-treated copper surfaces. Each copper surface underwent an oxygen plasma treatment for 1 min to activate functional groups on the surfaces and enhance cross-linking. SHMC immobilization was then applied to the pre-treated copper surfaces using the practical spray-coating method. The purpose of such a coating is to decrease the ice adhesion strength and prevent ice formation on the surface of the sample [28]. In this study, we aimed not only to detect ice formation, with the uncoated samples serving this purpose, but also to observe if there were any differences in ice formation between the coated and uncoated samples.

## 6. Experimental Set-Up

**Figure 5:**
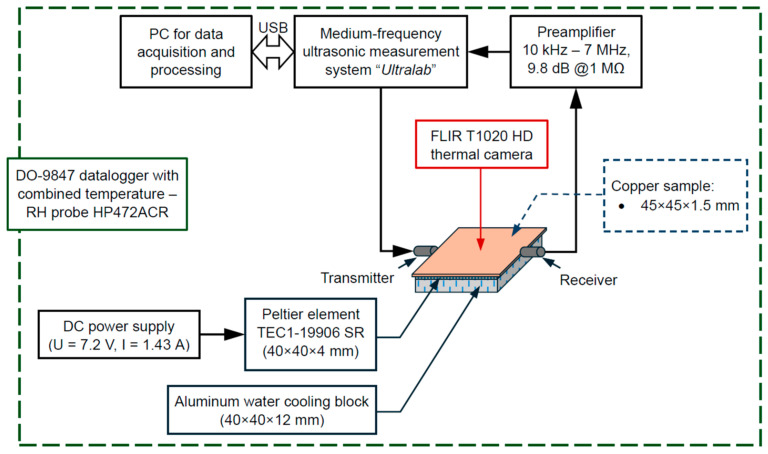
Schematic of the experimental set-up.

The ultrasonic characterization of the icing formation on the copper samples was performed using the experimental set-up presented in Figure 5. The measurements were performed using the medium-frequency ultrasonic measurement system “Ultralab”, developed at Kaunas University of Technology (Kaunas, Lithuania). The measurements were performed by arranging ultrasonic transducers in a trough transmission configuration, where one of them acts as a transmitter and another as a receiver. During the experimental investigation, ultrasonic transducers were attached to the edge of the copper plate, and signals were recorded during the period of plate cooling. Measurements were performed when the ultrasonic transducer was excited by a 1 period 2.25 MHz square pulse signal. The sample itself was attached to the cold plate of the Peltier cooler (loosely placed without using any adhesive material in between). The used Peltier element was a TEC1-19906 SR (40 × 40 × 4 mm) 24 V device rated at 6 A and with a cooling power of 120 W. A liquid cooling loop was used to reduce and maintain the temperature of the hot side of the Peltier element for thermal stability in the time-dependent icing experiments. The temperature of the samples during the ice formation was monitored using an IR thermal camera (FLIR T1020, FLIR Systems, Wilsonville, OR, USA) positioned over the samples under investigation. The temperature on the surface of the sample ranged from approx. 25 °C to −16 °C. The overall temperature and relative humidity in the room during the experimental investigation was monitored using a DO-9847 (Sanseca Italy Srl, Caselle di Selvazzano, Italy) datalogger with a HP472ACR combined temperature–RH probe. During the time of the experimental measurements, a temperature of 23.7 °C and an RH of 24.5% were observed inside the room.

## 7. Results and Discussion

**Figure 6:**
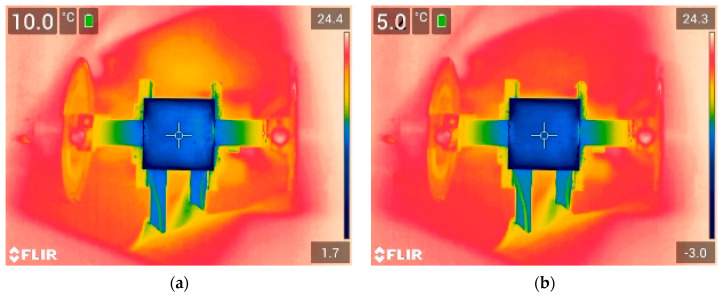

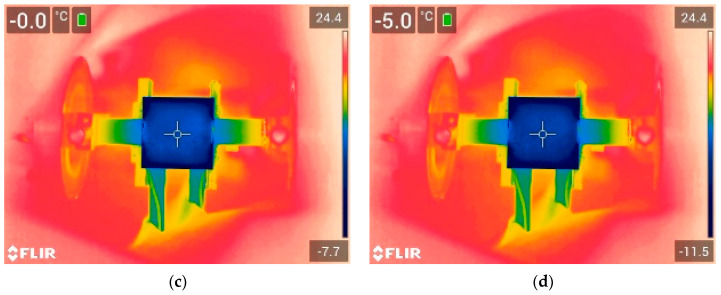
Icing process on the 1.5 mm thick uncoated copper sample, representing different surface temperatures at central position: (**a**) 10 °C, (**b**) 5 °C, (**c**) 0 °C, and (**d**) −5 °C.

**Figure 7:**
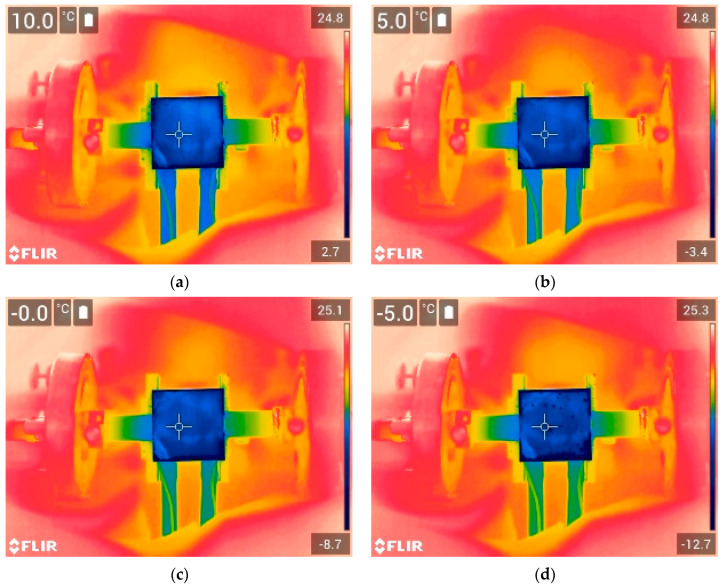
Icing process on the 1.5 mm thick coated copper sample representing different surface temperatures at central position: (**a**) 10 °C, (**b**) 5 °C, (**c**) 0 °C, and (**d**) −5 °C.

The thermographic images of the icing process observed with an IR camera on the uncoated and coated copper samples can be seen in Figure 6 and Figure 7, respectively. The images are presented at surface temperatures of 10 °C, 5 °C, 0 °C, and −5 °C at the marker position (center of the samples). It can be observed that the icing process was non-uniform, and the samples exhibited some thermal gradient, with the edges of the samples cooling down faster compared to the center point. For instance, when the temperature at the center of the sample reached 5 °C for the uncoated sample (Figure 6b), the edges of the sample were already below 0 °C, at approximately −3.0 °C. However, for the purpose of attaining a better understanding, we assumed that the temperatures measured at the marker position represented the overall surface temperature of the sample. Nevertheless, it should be noted that such a thermal gradient may impact wave propagation, and ice formation may begin even if the temperature reading at the center of the sample is positive.

**Figure 8:**
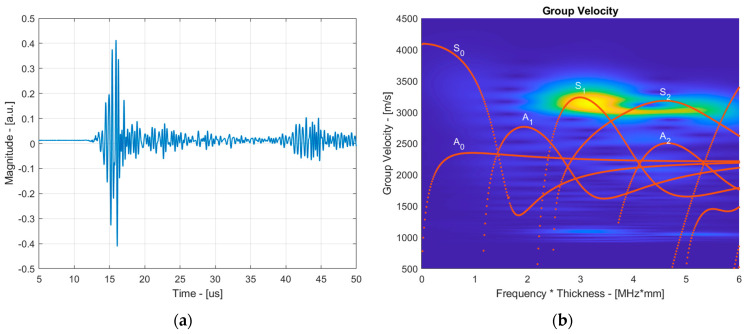
The time domain signal obtained on the 1.5 mm copper sample at 25 °C (**a**) and the reconstructed group velocity dispersion curves from the obtained signal (**b**).

To verify whether the S_1_ mode propagated in the structure and arrived first at the sensor, the group velocity reconstruction approach described in Section 4 was implemented. An example of the signal propagating through the copper sample at room temperature is presented in Figure 8a. This signal was filtered using a Gaussian filter, with the center frequency of the filter varying from 0 to 5 MHz with an increment of 0.1 MHz, and the group velocity’s dependence on the frequency was reconstructed considering a fixed propagation distance. The experimentally reconstructed group velocity dispersion curves can be seen in Figure 8b. For better understanding, the group velocity dispersion curves calculated with the SAFE method on the 1.5 mm copper sample at room temperature, shown in Figure 1b, are overlaid on the B-scan image. The results clearly indicate the presence of the S_1_ mode at a 3 MHz·mm frequency with a group velocity of approximately 3150 m/s. A footprint of the S_2_ mode can also be observed, which exhibited a group velocity similar to the S_1_ mode. Similar results were observed in the coated sample; however, to avoid repetition, they were omitted.

**Figure 9:**
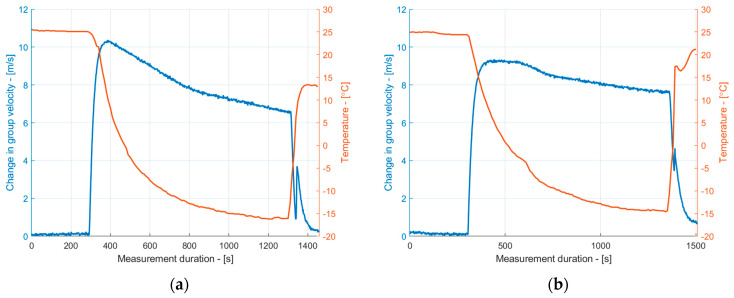
The dependence of the group velocity of the S_1_ mode on the temperature and ice formation for uncoated (**a**) and coated (**b**) copper samples.

The ice accretion on the surface of the sample was analyzed using the group velocity dependence of the S_1_ mode on the temperature. The dependencies obtained for the uncoated and coated samples are presented in Figure 9. To avoid the co-existing S_2_ mode, each signal was filtered with a Gaussian bandpass filter with a center frequency of 2.25 MHz and a bandwidth of 50% at a −6 dB level. From the results, it can be observed that as the temperature decreased, the group velocity of the S_1_ mode initially increased due to changes in the elastic properties of the material, as demonstrated in Figure 3. However, when the surface temperature reached approximately 5 °C, the group velocity of the S_1_ mode began to decrease due to the formation of the ice layer, primarily around the edges of the sample, as shown in Figure 6 and Figure 7. As the temperature continued to decrease, the group velocity steadily decreased as a result of ice accretion. This confirms the general trends presented in Figure 4.

**Figure 10:**
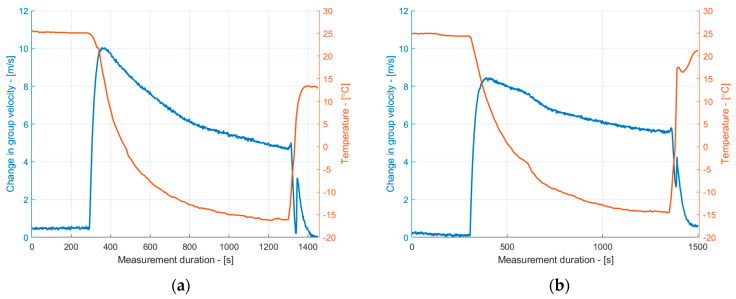
The dependence of the group velocity of the S_1_ mode on the temperature and ice formation for uncoated (**a**) and coated (**b**) copper samples, taking into the account the thermal expansion.

**Figure 11:**
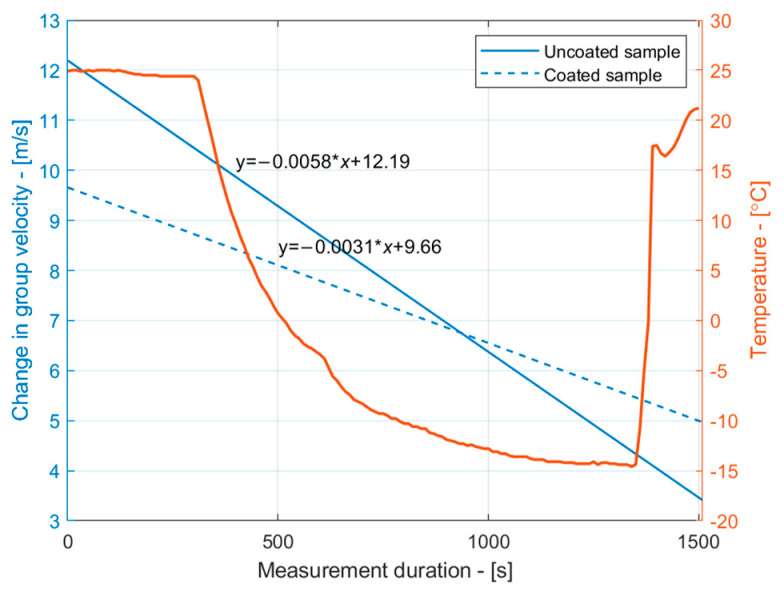
A linear approximation of the S_1_ mode group velocity change due to temperature for uncoated (solid line) and coated (dashed line) samples.

The overall changes observed in the S_1_ mode were relatively small, indicating around from 1.5 m/s to 3 m/s variations in the group velocity below 0 °C. To accurately account for such changes, the thermal expansion of the material must be taken into consideration. It is known that any material subjected to temperature fluctuations will undergo thermal expansion, resulting in modifications to its dimensions. In this study, the temperature ranged from 25 °C to −15 °C, potentially impacting the measurement results of the group velocity. To mitigate this effect, the linear thermal expansion of the copper samples was estimated according to the following equation:

(7) $$∆L=\alpha \cdot L_{b}\cdot ∆T,$$

where α is the linear thermal expansion coefficient (1/°C), and ∆T is the temperature change in °C. In this study, we used a 16.6 × 10^−6^/°C thermal expansion coefficient to describe copper. The obtained thermal expansion dependencies on the temperature revealed that the length of the samples decreased by approximately −0.03 mm per desired temperature range. Although the changes in the sample dimensions were negligible, they may contribute to a group velocity measurement error of up to 2 m/s, which is significant for early-stage ice detection. Therefore, the thermal expansion of the copper was taken into account in the reconstruction of the group velocity dependencies of the S_1_ mode on the temperature. The compensated group velocity dependencies are presented in Figure 10. The results in Figure 10 suggest that the slope of the group velocity decay changed. A linear approximation of the group velocity decay due to icing can be observed in Figure 11. It is apparent that the group velocity changes were more pronounced for the uncoated sample, suggesting faster ice accretion. On the other hand, surface coatings may alter the surface wetting conditions, and without measuring the actual thickness of the ice layer, it is impossible to conclude whether ice did not form on the coated surface at all. Nevertheless, the group velocity measurements show that the ice loading acted differently on the coated and uncoated samples.

**Figure 12:**
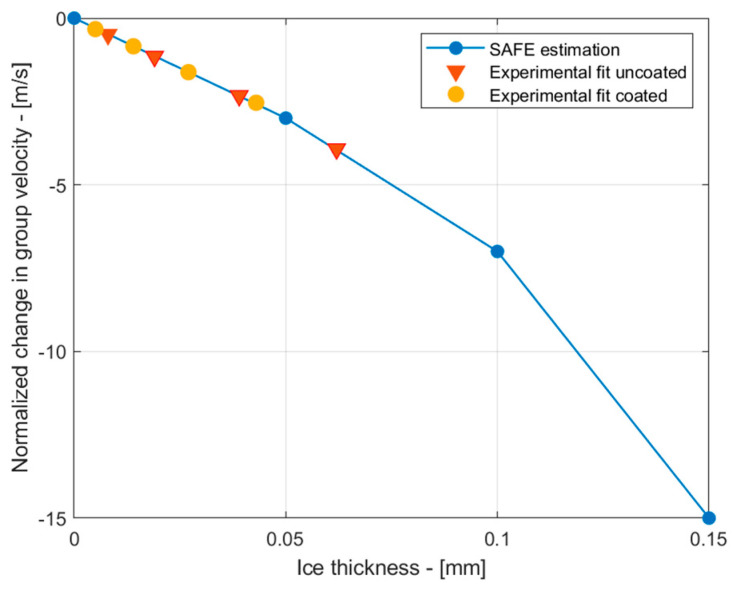
The theoretical decay of the group velocity of S_1_ versus thickness of the ice (blue) and the fitted experimental data (red).

Although the thickness of the ice layer itself was not measured during the experiments, the changes in the group velocity within the temperature for the uncoated sample were compared with theoretical predictions of the group velocity changes due to icing for the S_1_ mode. In these calculations, the group velocity of the S_1_ mode at different ice thicknesses up to 0.15 mm obtained from the SAFE method, as shown in Figure 4b, was used as reference data. The experimental group velocity readings were taken at temperature ranges between 5 °C and −15 °C, with decrements of 5 °C. In this analysis, the experimentally obtained results were synthetically fitted to the theoretical SAFE data in order to reconstruct the likely ice thickness. The results are presented in Figure 12. These results suggest that as the group velocity of the uncoated sample changed by less than 5 m/s, the likely thickness of the ice on the surface of the sample was below 7 µm. Since sprayed ice was not used to build up ice layers in our experiments and the ice condensed from the relative humidity of the air, it is assumed that the estimated ice thicknesses are credible. For the coated sample, the estimated likely thickness was even less, reaching up to 45 µm. This confirms the effectiveness of the coating, which aims to reduce the ice adhesion strength and its accretion.

## 8. Conclusions

This paper introduces a method for early-stage icing detection, with a focus on measuring the group velocity of the high-order S_1_ mode during the cooling cycle and correlating these values with the ice layer thickness. Two approaches to measure the group velocity of the S_1_ mode are proposed: a conventional method based on time-of-flight estimation for the direct measurement of the group velocity dependence on temperature and a 2D phase velocity dispersion reconstruction approach used to identify propagating modes using a single transmission signal. Using the latter approach, we confirmed the propagation of the S_1_ mode in the structure, while the former approach allowed us to estimate experimental group velocity dependencies on the temperature during the cooling cycle of copper samples, both for uncoated and coated samples with superhydrophobic multiscale anti-ice coatings.

The experimental results revealed that as the samples’ surface temperature decreased, the group velocity of the S_1_ mode increased due to changes in the material’s elastic properties. Below 5 °C, frost formation at the sample edges led to a subsequent decrease in the group velocity attributed to ice layer formation. To simulate early-stage ice formation, natural environmental humidity induced condensation on the sample surface, forming the ice layer. The experiments showed a distinct slope of the group velocity decay for the uncoated and coated samples, indicating that the coating prevented ice formation and reduced its adhesion. Finally, experimentally estimated group velocity changes due to ice formation were fitted to theoretical predictions of the SAFE method to inverse the actual ice thickness. The results demonstrated that the ice thickness increased up to 62 µm for the uncoated samples and up to 43 µm for the coated samples. These results highlight the technique’s ability to detect early-stage ice formation and to assess the effectiveness of anti-ice coatings.

## Figures and Tables

**Table 1:** Samples used for the detection of icing.

Sample No.	Substrate Material	Coated/Uncoated	Dimensions (mm)
(0865)	Copper	Uncoated	45 × 45 × 1.5
(0866)		SHMC-coated	45 × 45 × 1.5

## Data Availability

The data presented in this study are available on request from the corresponding author.

## References

[1] Green S. (2006). A Study of U.S. Inflight Icing Accidents and Incidents, 1978 to 2002. Proceedings of the 44th AIAA Aerospace Sciences Meeting and Exhibit.

[2] Aviation Safety Network (2017). West Wind Aviation ATR 42-300 Report.

[3] Aviation Safety Network (2019). Bek Air Flight 2100 Report.

[4] Deiler C., Fezans N. (2020). Performance-Based Ice Detection Methodology. J. Aircr..

[5] Maio L., Moll J., Memmolo V., Simon J. (2022). Ultrasonic Inspection for Ice Accretion Assessment: Effects on Direct Wave Propagation in Composite Media. Mech. Syst. Signal Process..

[6] Cao Y., Tan W., Wu Z. (2018). Aircraft Icing: An Ongoing Threat to Aviation Safety. Aerosp. Sci. Technol..

[7] Li S., Paoli R. (2022). Aircraft Icing Severity Evaluation. Encyclopedia.

[8] Parent O., Ilinca A. (2011). Anti-Icing and De-Icing Techniques for Wind Turbines: Critical Review. Cold Reg. Sci. Technol..

[9] Løw-Hansen B., Hann R., Stovner B.N., Johansen T.A. (2023). UAV Icing: A Survey of Recent Developments in Ice Detection Methods. IFAC-PapersOnLine.

[10] Yamazaki M., Jemcov A., Sakaue H. (2021). A Review on the Current Status of Icing Physics and Mitigation in Aviation. Aerospace.

[11] Shu L., Yu Z., Hu Q., Jiang X. (2023). Numerical and Experimental Investigations of Deicing Performance for the Pneumatic Impulse Deicing Method. J. Mar. Sci. Eng..

[12] Guo X., Yang Q., Zheng H., Dong W. (2024). Integrated Composite Electrothermal De-Icing System Based on Ultra-Thin Flexible Heating Film. Appl. Therm. Eng..

[13] Caliskan F., Hajiyev C. (2013). A Review of in-Flight Detection and Identification of Aircraft Icing and Reconfigurable Control. Prog. Aerosp. Sci..

[14] Schlegl T., Moser M., Loss T., Unger T. A Smart Icing Detection System for any Location on the Outer Aircraft Surface. Proceedings of the Conference: International Conference on Icing of Aircraft, Engines, and Structures.

[15] Zheng D., Li Z., Du Z., Ma Y., Zhang L., Du C., Li Z., Cui L., Zhang L., Xuan X. (2022). Design of Capacitance and Impedance Dual-Parameters Planar Electrode Sensor for Thin Ice Detection of Aircraft Wings. IEEE Sens. J..

[16] Mäder T., Nestler M., Kranz B., Drossel W. (2018). Studies on Sheet-Metal Compounds with Piezoceramic Modules for Icing Detection and De-Icing. Adv. Eng. Mater..

[17] Zou J., Ye L., Ge J. (2013). Ice Type Detection using an Oblique End-Face Fibre-Optic Technique. Meas. Sci. Technol..

[18] Ikiades A., Spasopoulos D., Amoiropoulos K., Richards T., Howard G., Pfeil M. (2013). Detection and Rate of Growth of Ice on Aerodynamic Surfaces using its Optical Characteristics. Aircr. Eng. Aerosp. Technol. Int. J..

[19] Liu Y., Chen W., Bond L.J., Hu H. A Feasibility Study to Identify Ice Types by Measuring Attenuation of Ultrasonic Waves for Aircraft Icing Detection. Proceedings of the ASME 2014 4th Joint US-European Fluids Engineering Division Summer Meeting collocated with the ASME 2014 12th International Conference on Nanochannels, Microchannels, and Minichannels.

[20] Fuleki D., Sun Z., Wu J., Miller G. (2017). Development of a Non-Intrusive Ultrasound Ice Accretion Sensor to Detect and Quantify Ice Accretion Severity. Proceedings of the 9th AIAA Atmospheric and Space Environments Conference.

[21] Sohail M., Pfeiffer H., Wevers M. (2022). Addressing Safety Concerns in Hybrid Electric Aircrafts: In-Flight Icing Detection, Moisture Detection in Fuselage and Electrical Wiring and Interconnect System (EWIS). IOP Conf. Ser. Mater. Sci. Eng..

[22] Gao H., Rose J.L. (2009). Ice Detection and Classification on an Aircraft Wing with Ultrasonic Shear Horizontal Guided Waves. IEEE Trans. Ultrason. Ferroelectr. Freq. Control.

[23] Mendig C., Riemenschneider J., Monner H.P., Vier L.J., Endres M., Sommerwerk H. (2018). Ice Detection by Ultrasonic Guided Waves. CEAS Aeronaut. J..

[24] Moll J., Simon J., Memmolo V. Surface Ice Detection on Composite Plates with Ultrasonic Guided Waves. Proceedings of the 2019 IEEE 5th International Workshop on Metrology for AeroSpace (MetroAeroSpace).

[25] Memmolo V., Moll J. (2020). Investigation on Guided Waves Propagation Across Ice Layers. Proc. SPIE.

[26] Tian Y., Duongthipthewa A., Chen Q., Guo H., Liu M., Zhang J., Zhou L. (2024). Quantitative Monitoring of Icing on CFRP Laminate with Guided Wave Combining Forward Modeling and Inverse Characterization. Struct. Health Monit..

[27] Muganlı Z., Saeidiharzand S., Rekuviene R., Samaitis V., Jankauskas A., Koşar A., Gharib G., Sadaghiani A. (2023). Development and Implementation of Microbial Antifreeze Protein Based Coating for Anti-Icing. Adv. Mater. Interfaces.

[28] Saeidiharzand S., Sadaghiani A.K., Yürüm A., Koşar A. (2023). Multiscale Superhydrophobic Zeolitic Imidazolate Framework Coating for Static and Dynamic Anti-Icing Purposes. Adv. Mater. Interfaces.

[29] Yule L., Zaghari B., Harris N., Hill M. (2021). Modelling and Validation of a Guided Acoustic Wave Temperature Monitoring System. Sensors.

[30] Boso D. (2013). A Simple and Effective Approach for Thermo-Mechanical Modelling of Composite Superconducting Wires. Supercond. Sci. Technol..

[31] Poots G., Makkonen L. (2000). Models for the Growth of Rime, Glaze, Icicles and Wet Snow on Structures. Philos. Trans. R. Soc. London.Ser. A Math. Phys. Eng. Sci..

[32] Tumsys O. (2022). Experimental Method for Simultaneous Determination of the Lamb Wave A0 Modes Group and Phase Velocities. Materials.

